# 

**Published:** 1906-03-15

**Authors:** 


					﻿Vol. LX.	MARCH 15, 1906.	No. 3,
THE
DENTAL REGISTER
EDITED BY/
NELVILLE S. HOFF, D.D(S.,
ANN ARBOR, l^ICH. /
PRICE, ONE DOLLAR A YEAR IN ADVANCE.
Published Monthly by
SAMUEL A. CROCKER & CO.,
Ohio Dental and Surgical Depot,
35 to 39 W. 5th St., Cincinnati, 0.
Entered at the Postoffice at Cincinnati, O., as second-class mail matter.
				

